# Exposure to drinking water pollutants and non-syndromic birth defects: a systematic review and meta-analysis synthesis

**DOI:** 10.1136/bmjopen-2024-084122

**Published:** 2024-11-11

**Authors:** Eric Jauniaux, Lydia Jeremiah, Biba Richardson, Ewelina Rogozińska

**Affiliations:** 1 EGA Institute for Women Health, University College London, London, UK; 2 Women’s Health Research Unit, Barts and the London School of Medicine and Dentistry, Queen Mary University of London, London, UK; 3 The EVIE Evidence Synthesis Research Group, Institute of Clinical Trials and Methodology, Faculty of Population Health Sciences, University College London, London, UK

**Keywords:** Fetal medicine, Maternal medicine, Prenatal diagnosis

## Abstract

**Abstract:**

**Objectives:**

To evaluate the association between drinking water pollutants and non-syndromic birth defects.

**Design:**

Systematic review and meta-analysis synthesis.

**Data sources:**

A search of MEDLINE, EMBASE and Google Scholar was performed to review relevant citations reporting on birth defects in pregnancies exposed to water pollutants between January 1962 and April 2023.

**Eligibility criteria:**

Prospective or retrospective cohort, population studies and case–control studies that provided data on exposure to drinking water pollutants around conception or during pregnancy and non-syndromic birth defects. We included studies published in the English language after the Minamata Bay disaster to reflect on contemporary concerns about the effect of environmental pollution and obstetric outcomes.

**Data extraction and synthesis:**

Two reviewers independently read the retrieved articles for content, data extraction and analysis. The methodological quality of studies was assessed using the Newcastle-Ottawa Scale. Included studies were assessed for comparability when considered for meta-analysis.

**Results:**

32 studies met inclusion criteria including 17 cohorts (6 389 097 participants) and 15 case–control studies (47 914 cases and 685 712 controls). The most common pollutants investigated were trihalomethanes (11 studies), arsenic (5 studies) and nitrates (4 studies). The studies varied in design with different estimates of exposure, different stages of gestation age and different durations of exposure to pollutants. 21 articles reported data on any birth defects in their population or study groups and the others on specific birth defects including congenital heart defects, neural tube defects, orofacial defects and hypospadias. An increased risk or higher incidence of overall birth defects was reported by 9 studies and for specific birth defects by 14 studies. Eight studies compared the risk or incidence of birth defects with exposure to different concentrations of the pollutants. The analysis showed an association between higher levels of trihalomethanes (TTMs) and arsenic increase in major birth defects (lower vs higher exposure (OR 0.76, 95% CI 0.65 to 0.89; p<0.001 and OR 0.56, 95% CI 0.39 to 0.82; p<0.005, respectively).

**Conclusion:**

The evidence of an association between exposure to average levels of common drinking water chemical pollutants during pregnancy and an increased risk or incidence of birth defects is uncertain. Available evidence indicates that some common chemical pollutants currently found in drinking water may have a direct teratogenic effect at high maternal exposure, however, wide variation in methodology limits the interpretation of the results. Future prospective studies using standardised protocols comparing maternal levels during all three trimesters of pregnancy and cord blood levels at birth are needed to better understand the placental transfer of water pollutants and accurately evaluate individual fetal exposure to drinking water pollutants.

**PROSPERO registration number:**

CRD42018112524.

**STRENGTHS AND LIMITATIONS OF THIS STUDY**

- This is the largest systematic review examining the possible association between known common drinking water pollutants, different drinking water pollutants and non-syndromic birth defects using an a priori designed protocol registered on an international register of systematic reviews.
- The systematic review only included studies that provided secure medical records, regional or national databases with detailed descriptions of all birth defects in a defined population with detailed pathology record during the study period.
- We included studies that were published since the Minamata Bay disaster to reflect on contemporary concerns about the effect of environmental pollution and obstetric outcomes.
- The main limitation of this study is the many challenges in assessing prenatal exposure to specific chemical and toxics at different dosages and different gestation ages.
- The studies included in our systematic review had varied study designs, including differences in timing and duration of exposures to a drinking water pollutant before and during pregnancy, different methodologies to evaluate the concentration of each pollutant component, and different ranges and regulatory limits for an individual pollutant level between countries, limiting the extend of the meta-analysis and interpretation of our results.

## Introduction

 Unlike other commodities, water is paramount for human survival and only 0.4% of the water on earth is fresh water readily available for consumption.^1^ Industrial methods, such as fossil fuel extraction, chemical waste treatment and agricultural processes, have threatened freshwater ecosystems for decades.^2^ More recently, climate change has been shown to have a disproportionate effect on pregnant women’s health, directly through exposure to toxic chemicals and vectorborne diseases and indirectly by influencing food and water security.^3 4^ These effects are further exacerbated in low-resourced countries (LRCs) where prenatal and maternity healthcare is limited. Warmer temperatures also increase the environmental distribution and toxicity of chemical pollutants including air pollutants, persistent organic pollutants, such as some organochlorine pesticides and other classes of pesticides.^5^ The effects of water pollution on aquatic biota and ecosystems, and in particular, on fish reproduction and survival in lakes and rivers further compromise the food chain in LRCs.^6^

Unlike air pollution, in which only a small handful of parameters need tracking, thousands of water quality parameters have been identified by organisations such as the WHO.^7^ The range of pollutants found in drinking water is ever-increasing and now includes pharmaceutical by-products such as hormones, painkillers and antibiotics,^8^ personal care products^9^ and drugs of abuse.^10^ Chemical contaminants and disinfection by-products (DBP) found in drinking water have been associated with adverse pregnancy outcomes including fetal growth restriction, premature delivery and stillbirth.^11^ The environmental disaster of Minamata Bay in the late 1950s,^12^ where children whose mothers had eaten excessive amounts of fish and shellfish contaminated by methylmercury during pregnancy had neurological defects from early in life, was pivotal in highlighting the relationship between maternal exposure to water pollution and the developmental anomalies. However, this event has been largely forgotten and eclipsed by pharmaceutical drug disasters (thalidomide, diethylstilbestrol). There are currently limited data on chemical water pollution and the risks of birth defects.^13^

The pathogenesis of congenital anomalies in humans and other mammals is multifactorial, caused by complex interactions between genes and environment during the organogenesis phase of fetal development.^14^ Water pollutants can have a preconceptional mutagenic and postconceptional teratogenic action, periconceptional endocrine disruption and epigenetic effects. The objective of this study was to systematically review the literature to evaluate the possible association between pollutants found in drinking water and different non-syndromic birth defects.

## Methods

### Data sources and search strategy

This systematic review was guided by a prospectively developed protocol registered with the International Prospective Register of Systematic Reviews (PROSPERO number CRD42018112524). We searched MEDLINE, EMBASE and Google Scholar with a search strategy including the following MeSH (Medical Subject Headings) terms: “drinking water“ OR “water pollution“ OR “water toxicant“ OR “water pollutant“ OR “pesticides” OR “fertilisers” OR “microplastics” OR “lead” OR “mercury” AND “birth defects” OR “congenital malformations” OR “fetal anomalies”. The database was searched from January 1958 to April 2023. Additional studies were identified from reference lists of full-text articles for relevant citations, expert reviews and editorials. The study is reported in accordance with Preferred Reporting Items for Systematic Reviews and Meta-Analysis.^15^ The search was limited to human studies and articles published in English (online supplemental appendix 1).

### Selection criteria and data extraction

Two reviewers (LJ and BR) independently assessed identified titles and abstracts against the eligibility criteria. The study had to meet the following criteria to be included in the review: (1) observational study (population, cohort or case control) that investigates the association between non-syndromic birth defects and exposure to one or more drinking water pollutants around the time of conception and/or during pregnancy; (2) study had to report outcomes with appropriate estimates of women who were or became pregnant during the study and newborns born during the study period. We excluded letters, editorials, case reports and duplications of previously published data from the same centres or with an indication of overlapping in the methodology (online supplemental appendix 1). As we aimed to evaluate the incidence of non-syndromic birth defects according to drinking water pollutants in observational studies, we also excluded case–control studies including syndromic birth defects or using syndromic birth defects as controls, studies that did not report on contemporary measurements of a specific pollutant from the area under investigation and studies that did not describe the timing of exposure before or during pregnancy.

Two reviewers (LJ and BR) independently assessed the content of the full text for content, and subsequently extracted relevant data. The extracted data were checked again by two researchers (EJ and ER) and any discrepancies were resolved between the reviewers through discussion. Data from eligible studies were entered into an Excel spreadsheet including the first author of the study, year of publication, country of origin, study characteristics (study design, sample size, recruitment setting and pollutant(s) investigated), methodology and outcomes of interest.

### Quality assessment

The quality of eligible studies was independently assessed by LJ and EJ using the Newcastle-Ottawa Quality Assessment Scale.^16^ Each type of study was evaluated on three domains: selection of study groups, comparability of groups and ascertainment of exposure (for case control) or outcome (for cohort). Each positive criterion scores 1 point, except comparability, which scores up to 2 points. A score of 7–9 was considered low, 4–6 moderate and 0–3 high risk of bias.

### Data synthesis

Included studies were assessed for comparability when considered for meta-analysis. Studies that only reported adjusted measures of association that is, risk ratio, OR or log OR, were not included in the meta-analysis. Due to a low number of available studies per type of pollutant, we used a fixed effect model to pool data where possible. Between-study heterogeneity was assessed using I^2^ statistic. Analyses were conducted by using Review Manager (V.5.4.1).

### Patient and public involvement

None.

## Results

### Search results

**Figure 1:**
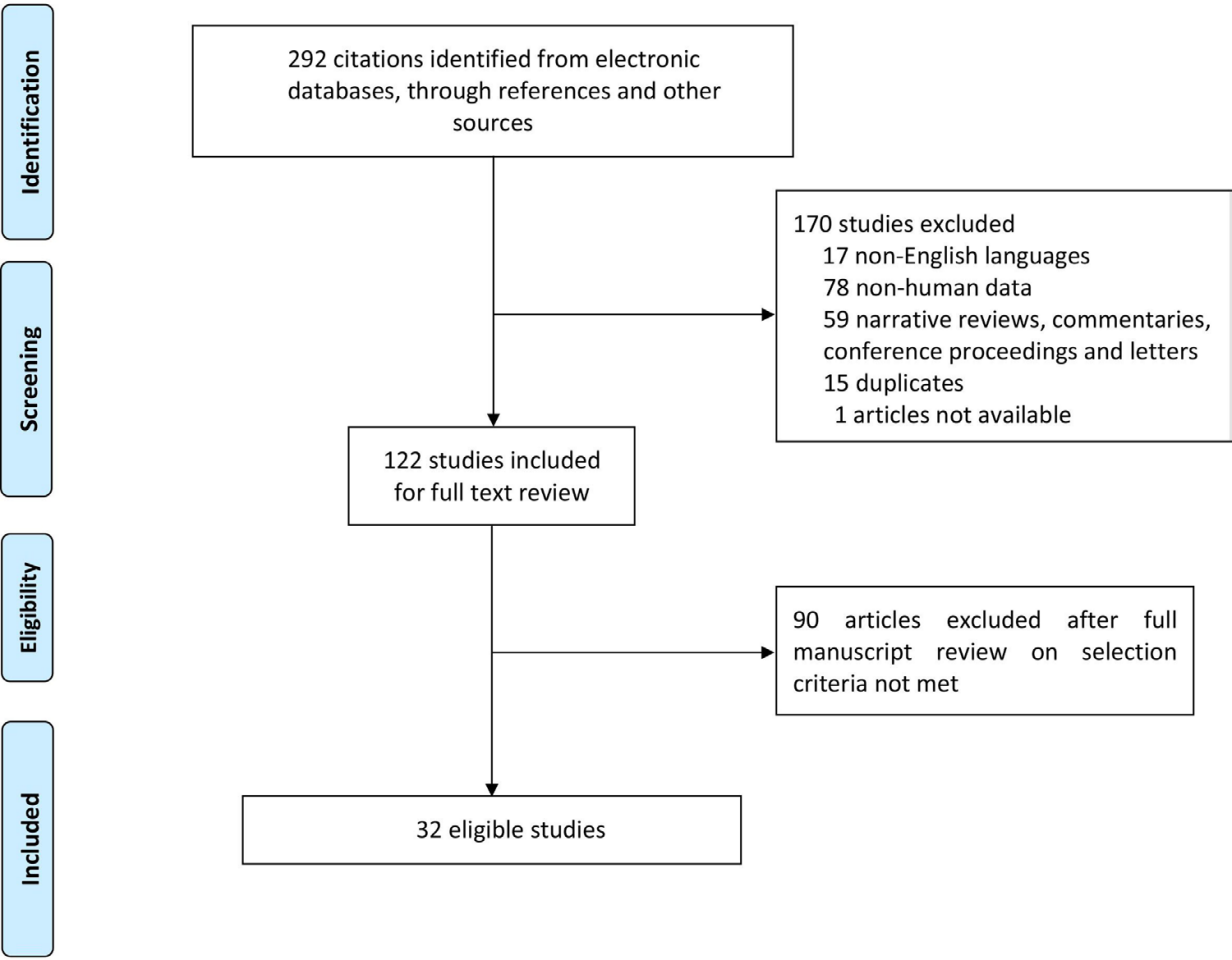
Flow diagram for study selection.

The search identified 292 potential citations. From these, 170 were excluded after reviewing the title and abstract. Following full-text reading, 32 articles were included in the final analysis (6 389 097 participants in the cohort studies and 47 914 cases with a birth defect or exposed to one or more drinking water pollutants and 685 712 controls). The process of selection of the articles is summarised in figure 1.

### Characteristics of studies included in the systematic review

The included 32 studies^17^,^48^ were conducted in 12 different countries over a period of 72 years and published between 1984 and 2022 (table 1).

**Table 1:** Characteristics of included studies

Author *et al* (year)	Country	Study period	Study design	Population studied (n)	Pollutant(s) investigated
Dorsch *et al* (1984)^17^	Australia	1951–1979	Retrospective case control	218 cases with a BD218 normal controls	Nitrates
Zierler *et al* (1988)^18^	USA	April 1980–March 1983	Retrospective case control	270 cases with CHDs665 normal controls	Arsenic, barium, cadmium, chromium, lead, mercury, selenium, silver, fluoride, nitrates and sodium
Deane *et al* (1989)^19^	USA	1980–1981	Retrospective case control	191 cases exposed210 non-exposed controls	Trichloroethane*
Donald Whorton *et al* (1989)^20^	USA	1978–1982	Retrospective cohort	46 328 live births	DBCP
Swan *et al* (1989)^21^	USA	1980–1985	Retrospective cohort	65 704 live births	Trichloroethane
Wrensch *et al* (1990)^22^	USA	1980–1985	Retrospective cohort	1480 pregnancies	Trichloroethane
Bove *et al* (1995)^23^	USA	1985–1988	Retrospective cohort*	81 523 births (including 594 stillbirths)	THMs
Macdonell *et al* (2000)^24^	UK	1983–1995	Retrospective cohort	144 006 live births	Lead
Dodds *et al* (2001)^25^	Canada	1988–1995	Retrospective cohort	49 842 live births	THMs†
Shaw *et al* (2003)^26^	USA	June 1989–May 1991	Retrospective case control	803 cases with a BD1020 normal controls	THMs
Brender *et al* (2006)^27^	USA	March 1995–May 2000	Retrospective case control	184 cases with NTDs225 normal controls	Arsenic, cadmium, lead and mercury
Kwok *et al* (2006)^28^	Bangladesh	2002–2003	Retrospective cohort	2189 pregnancies	Arsenic
Chisholm *et al* (2008)^29^	Australia	2000–2004	Retrospective cohort	20 870 births and TOPs	THMs
Hwang *et al* (2008)^30^	Taiwan	2001–2003	Retrospective cohort	396 049 births	THMs
Nieuwenhuijsen *et al* (2008)^31^	UK	1998–2001	Retrospective cohort	2 605 226 births and TOPs	THMs
Aschengrau *et al* (2009)^32^	USA	1969–1983	Retrospective case control	1658 cases exposed2999 non-exposed controls	PCE‡
Righi *et al* (2012)^33^	Italy	2002–2005	Retrospective case control	1150 cases with a BD4984 normal controls	THMs
Stassen *et al* (2012)^34^	Bolivia	2006	Retrospective cohort	212 pregnant patients	Cadmium and lead
Brender *et al* (2013)^35^	USA	March 1995–May 2000	Retrospective case control	3159 cases with a BD1551 normal controls	Nitrates
Grazuleviciene *et al* (2013)^36^	Lithuania	2007–2009	Prospective cohort	3074 live births	THMs
Sanders *et al* (2014)^37^	USA	2003–2008	Retrospective case control	20 151 cases with a BD668 381 normal controls	Arsenic, cadmium, manganese and lead
Mazumdar *et al* (2015)^38^	Bangladesh	2013 (April–November)	Retrospective case control	57 cases with NTDs55 normal controls	Arsenic
Winston *et al* (2016)^39^	USA	1998–2005	Retrospective case control	343 cases of hypospadias1422 normal controls	Atrazine
Kim *et al* (2017)^40^	USA	1999–2005	Retrospective case control	18 291 cases a BD4414 normal controls	Atrazine
Marie *et al* (2018)^41^	France	2003, 2006, 2010	Retrospective cohort	5263 pregnancies	Arsenic
Suhl *et al* (2018)^42^	USA	2000–2011	Retrospective case control	435 cases of OFDs1267 normal controls	Arsenic
Weyer *et al* (2018)^43^	USA	2000–2005	Retrospective case control	680 cases of OFDs1826 normal controls	THMs
Blaisdell *et al* (2019)^44^	USA	2004–2008	Retrospective cohort	348 250 live births	Nitrates
Zaganjor *et al* (2020)^45^	USA	2000–2005	Retrospective case control	324 cases of hypospadias889 normal controls	THMs
Richter *et al* (2021)^46^	Denmark	1997–2014	Retrospective cohort	1 042 413 live births	Arsenic
Säve-Söderbergh *et al* (2021)^47^	Sweden	2005–2015	Prospective cohort	623 468 newborns	THMs
Stayner *et al* (2022)^48^	Denmark	1991–2013	Retrospective cohort	1 018 914 live births	Nitrates

* Trichloroethane is also known as methyl chloroform or chlorothene.

† Common Trihalomethanes (THMs) include fluorofrom, chloroform, dichloromethane (BCDM), halocetic acidhaloacetic acids () and bromoform).

‡ Tetrachloroethylen, Ttrichloroethylene () and THMs are part of Volatile organic compounds (); ; ; ; defects; ; ; OFDs: Orofacial defects; of pregnancy; defects.

BCDMbromodichloromethaneBDsbirth defectsCHDscongenital heart defectsDBCPdibromochloropropaneNTDsneural tube defectsOFDsorofacial defectsPCEtetrachloroethyleneTHMstrihalomethanesTOPtermination of pregnancy

Only seven of the studies were published before the year 2000.^17^,^23^ 15 studies had a case–control design,^17^,^1926 27 32 33 35 37^ the others were cohort studies. The 15 case–control studies compared cases presenting with a non-syndromic birth defect at delivery with normal controls^1718 26 27 33 35 37^,^40 42 43 45^ or cases exposed to one or more drinking water pollutants with non-exposed controls.^19 32^ All case–control studies, except one,^17^ used unequal numbers of cases and controls with one study comparing 20 151 cases with a birth defect with 668 381 normal controls.^37^ All studies, except one,^47^ were retrospective. 11 studies analysed data following maternal exposure to trihalomethanes (THMs)^2325 26 29^,^31 36 43 45^ in drinking water, five studies reported on arsenic,^28 38 41 42 46^ four on nitrates,^17 35 44 48^ three on trichloroethane,^19 21 22^ two on atrazine,^39 40^ one on tetrachloroethylene (PCE), one on dibromochloropropane and one on lead.^24^ Four studies investigated more than one different drinking water pollutants.^18 27 33 37^

19 studies used the data on one or more chemicals provided by the local water providers matched with individual home addresses,^1718 20^,^22 25^ three from a national water provider database^46^,^48^ and one from a national water provider database and from local water measurements (table 2).^41^

**Table 2:** Methodology and main outcomes of included studies

Author *et al* (year)	Exposure evaluation	Timing of exposure	Type of congenital anomaly recorded	Main findings
Dorsch *et al* (1984)^17^	Local water provider data, individual household address and interview on water consumption	1st trimester	Major BDs	Increased risk (RR 4.1, 95% CI 1.7; 10.0) of overall BDs in groundwater compared with rainwater subgroup with nitrates exposure.
Zierler *et al* (1988)^18^	Local water provider data and individual birth certificate address	Conception date	CHDs	No association (PR 1.0, 95% CI 0.71; 0.89) found for the overall risk of BDs but increased risk (PR 3.4, 95% CI 1.3; 8.9) of coarctation of the aorta with arsenic exposure.
Deane *et al* (1989)^19^	Water sample measurements in exposed area and individual household address	Pregnancy	Major BDs	Increased risk (RR 3.1, 95% CI 1.1; 10.4) of overall BDs with trichloroethane exposure.
Donald Whorton *et al* (1989)^20^	Local water provider data and individual birth certificate address	Pregnancy	Major BDs	No association (aRR 0.65, 95% CI 0.71; 0.94) between the overall risk of BDs and DBCP exposure.
Swan *et al* (1989)^21^	Local water provider data, individual household address and interview on water consumption	1st trimester	CHDs	Increased risks (RR 2.2, 95% CI 1.2; 4.0) of CHDs with trichloroethane exposure.
Wrensch *et al* (1990)^22^	Local water provider data, individual household address and interview on water consumption	1st trimester	Major BDs	Non-significant increase in the incidence (OR 1.4, 95% CI 0.90; 2.1) of overall BDs with trichloroethane exposure.
Bove *et al* (1995)^23^	Water sample measurements in local area and birth certificate address	Pregnancy	Major BDs	Higher incidence of overall BDs (OR 1.57, 50% CI 1.42; 1.75) with THMs exposure.
Macdonell *et al* (2000)^24^	Water sample measurements from individual household address	Time of birth	NTDs	No association was found between prevalence of NTDs/1000 livebirths and lead exposure.
Dodds *et al* (2001)^25^	Local water provider data and individual household address	First and second month	Major BDs	Increased risk (aRR 2.5, 95% CI 0.67; 2.10) of NTDs, no association (aRR 1.01, 95% CI 1.2; 5.1) with OFDs and decreased risk (aRR 0.3, 95% CI 0.2; 0.7) of CHDs with BDCM exposure.
Shaw *et al* (2003)^26^	Local water provider data and individual household address	3 months before conception and 1st trimester	NTDs	No association (aOR 0.9, 95% CI 0.85; 0.97)* found between NTDs and THMs exposure.
Brender *et al* (2006)^27^	Local water provider data, individual household address, interview on water consumption and work-related exposure	Date of conception	Major BDs	Higher incidences of NTDs with arsenic exposure (OR 2.0, 95% CI 0.1; 3.1) and mercury exposure (OR 2.0, 95% CI 0.3; 15.2) but not for lead exposure (OR 0.8, 95% CI 0.2; 2.6).
Kwok *et al* (2006)^28^	Measured arsenic levels from individual household address and interview on water consumption	Pregnancy	Major BDs	Higher incidence (aOR 1.005, 95% CI 1.001; 1.010) of overall BDs with arsenic exposure.
Chisholm *et al* (2008)^29^	Water sample measurements and individual household address	Pregnancy and TOPs	Major BDs	Higher incidences of overall BDs (OR 1.22, 95% CI 1.01; 1.48) and CHDs (OR 1.62, 95% CI 1.04; 2.51) with THMs exposure.
Hwang *et al* (2008)^30^	Local water provider data and individual household address	1 month after conception	Major BDs	Higher incidences of VSDs (aOR1.81, 95% CI 0.98; 3.35), cleft palate (aOR 1.56, 95% CI 1.00; 2.41) and anencephaly (aOR 1.96, 95% CI 0.94; 4.07) with THMs exposure.
Nieuwenhuijsen *et al* (2008)^31^	Local water provider data and individual household address	1st trimester	Major BDs	Higher incidences of VSDs (OR 1.43, 95% CI 1.00; 2.04) with THMs exposure and major CHDs (OR 1.18, 95% CI 1.00; 1.39) and gastroschisis (OR 1.38, 95% CI 1.00; 1.92) with bromoform exposure.
Aschengrau *et al* (2009)^32^	Local water provider data and individual household address	Maternal LMP	Major BDs	Higher incidence (aOR 1.5, 95% CI 0.9; 2.5) of overall BDs with PCE exposure.
Righi *et al* (2012)^33^	Local water provider data and individual household address	1st trimester	Major BDs	Higher incidence (aOR 2.00, 95% CI 1.05; 3.82) of UGDs with chlorite exposure. No association between overall BDs with THMs and chlorate exposure.
Stassen *et al* (2012)^34^	River water sample measurements, hair sampling, individual household address and interview on water consumption	Pregnancy	Major BDs	No association (combined aOR 2.6, 95% CI 0.7; 9.2) between overall risk of BDs and cadmium or lead exposure.
Brender *et al* (2013)^35^	Water sample measurements from local drinking water distribution system, individual household address and interview on water consumption	1st trimester	Major BDs	Higher incidences of limb defects (aOR 1.79, 95% CI 1.05; 3.08), spina bifida (aOR 2.02; 95% CI 1.27; 3.22) and OFDs (aOR 1.45, 95% CI 1.10; 1.92) with nitrates exposure.
Grazuleviciene *et al* (2013)^36^	Water sample measurements from local drinking water distribution system and geocoded individual household address	Pregnancy	Major BDs	Higher incidence (aOR 2.16, 95% CI 1.05; 4.46) of overall BDs with BDCM exposure in the first month of pregnancy. No association was found between CHDs (aOR 1.54, 95% CI 0.89; 2.68) musculoskeletal defects (aOR 0.74, 95% CI 0.39; 1.42) and UGDs (aOR 3.01, 95% CI 1.11; 8.16) with THM exposure.
Sanders *et al* (2014)^37^	Local water provider data and individual household address	Pregnancy	Major BDs	Higher incidence (PR 1.6, 95% CI 1.1; 2.5) of conotruncals CHDs with manganese exposure but not for arsenic, cadmium and lead.
Mazumdar *et al* (2015)^38^	Well water sample measurements, maternal blood level of arsenic and individual household address	1st trimester	NTDs	No association (aOR 1.03, 95% CI 0.55; 1.91) between myelomeningocele and arsenic exposure.
Winston *et al* (2016)^39^	Local water provider data, estimate level in wells and individual household address	6–16 weeks of gestation	Hypospadias	No association (OR 1.0, 95% CI 0.97; 1.03) between hypospadias and atrazine exposure.
Kim *et al* (2017)^40^	Local water provider data and individual household address	Pregnancy	CHDs	No association (aOR 0.84, 95% CI 0.66; 1.06) between CHDs and atrazine exposure.
Marie *et al* (2018)^41^	Data from the national water database, water sample measurements and individual household address	12 months before conception and entire pregnancy	Major BDs	Higher incidence of overall BDs (aOR 2.41, 95% CI 1.36; 4.14) and CHDs (aOR 3.66, 95% CI 1.62; 7.64) with arsenic exposure in female newborns.
Suhl *et al* (2018)^42^	Local water provider data, individual household address and interviews on water consumption	3 months before conception and entire pregnancy	OFDs	No association (OR 0.9, 95% CI 0.4; 2.3) between OFDs and arsenic exposure.
Weyer *et al* (2018)^43^	Local water provider data, water sample measurements, individual household address and interview on water consumption	1 month before conception and 1st trimester	OFDs	No association between OFDs with THMs (OR 0.9, 95% CI 0.7; 1.3) or HAAs (OR 0.9, 95% CI 0.6; 1.4) exposure
Blaisdell *et al* (2019)^44^	Local water provider data and individual household address	12 months before conception and 1st trimester	Major BDs	Increased risk of limb defects (RR 1.26, 95% CI 1.05; 1.51) but not for OFDs (RR 0.93, 95% CI 0.82; 1.06), hypospadias (RR 0.98, 95% CI 0.91; 1.06), NTDs (RR 1.03, 95% CI 0.84; 1.27) or gastroschisis (RR 0.94, 95% CI 0.76; 1.16) with nitrate exposure.
Zaganjor *et al* (2020)^45^	Local water provider data, water sample measurements, individual household address and interview on water consumption	1 month before conception and 1st trimester	Hypospadias	No association between hypospadias and THMs (aOR 0.9, 95% CI 0.5; 1.4) or total HAAs (aOR 0.8, 95% CI 0.5; 1.4) exposure.
Richter *et al* (2021)^46^	National water provider database and individual household address	4th week of gestation	CHDs	Higher incidence of CHDs (aOR 1.42, 95% CI 1.24; 1.62) with arsenic exposure.
Säve-Söderbergh *et al* (2021)^47^	National water provider database and individual household address	1st trimester	Major BDs	Higher incidence of NTDs (OR 1.82, 95% CI 1.07; 3.12), UGDs (OR 2.06, 95% CI 1.53; 2.78), genitals (OR 1.77, 95% CI 1.38; 2.26) and limbs defects (OR 1.34, 95% CI 1.10; 1.64) lower incidence of CHDs (OR 0.87, 95% CI 0.77; 0.99) with THMs exposure.
Stayner *et al* (2022)^48^	National water provider database and individual household address	90 days before conception and pregnancy	Major BDs	No association (OR 0.93, 95% CI 0.88; 0.99) between overall BDs but higher incidence (OR 1.29, 95% CI 1.00; 1.66) of eye defects with nitrate exposure.

* adjusted for nitrate exposure (Croen 2001) adjusted for nitrate exposure^73^

aOR, adjusted OR; BDs, birth defects; CHDs, congenital heart defects; DBCP, dibromochloropropane; HAAs, haloacetic acids; LMPlast menstrual periodNTDs, neural tube defects; OFDsorofacial defectsPCE, tetrachloroethylene; ppb, parts per billion; PR, prevalence ratio; RR, relative riskTHM, trihalomethanes; UGDs, urogenital defects; VSDs, ventricular septal defects

The remaining studies used data on chemical exposure obtained directly from measurements in local drinking water distribution system^1923 34^,^36^ or from individual household including from common and private wells.^24 28 38^ In one study, the authors obtained data on the concentrations of arsenic in maternal blood^38^ and in another, lead level was measured in maternal hair.^34^ In 10 studies, the authors also obtained data from individual household consumption via interviews and/or questionnaires.^17 21 22 27 28 34 35 42 43 45^ In one of these studies, the authors also collected data from work-exposure to the pollutant.^27^ The timing of exposure to the pollutants was the first trimester of pregnancy in eight studies.^17 21 22 31 33 35 38 47^ The other studies used the timing of exposure ranging from 12 months before conception to the time of birth (table 2). 21 articles reported data on any birth defects in their population or study groups^1719 20 22 23 26^,^37 41 44 47 48^ and the others on specific birth defects including congenital heart defects (CHDs),^18 21 40 46^ neural tube defects (NTDs),^24 26 38^ orofacial defects (OFDs)^42 43^ and hypospadias.^39 45^ An increased risk or higher incidence of overall birth defects was reported by nine studies^17 19 23 28 29 32 33 36 41^ and for specific birth defects by 14 studies.^1821 25 27 29^,^31 35 37 41 44 46^ The remaining studies found no association between one or more pollutants and one or more birth defects. In one study, the authors^26^ adjusted their results for published nitrate levels from the same study groups.

### Assessment of study quality

The studies were rated based on selection, comparability and outcome ascertainment. Overall, 5 studies had a low risk of bias and 18 studies had moderate risk of bias (online supplemental figure 1).

### Data synthesis

The detailed outcomes of cohort studies according to overall birth defects and individual organ systems for THM (n=7), arsenic (n=3) and nitrates (n=2) are presented in online supplemental table 1. The risk or incidence of birth defects to the corresponding drinking water pollutants was categorised according to the average water level,^23^ different concentrations^2528^,^30 36 46 47^ or below or above the limit of a National Environment Agency.^41 44 48^ Out of the eight studies comparing the risk or incidence of birth defects with exposure to different concentrations of the pollutants, five used three categories (low–medium–high)^29^,^3136 47^ and three used four or more different concentration ranges.^25 28 46^ In those studies using >3 level ranges, the authors compared the highest with the lowest levels or levels below the detection level of the assay. All authors, except one,^29^ adjusted their analysis for standard potential confounders including maternal age, body mass index, fetal gender and parity. In addition, nine authors included socioeconomic background and/or education status,^2325 28 31 41 44 46^,^48^ four included maternal smoking^25 44 47 48^ and two maternal gestational diabetes.^30 47^

**Figure 2:**
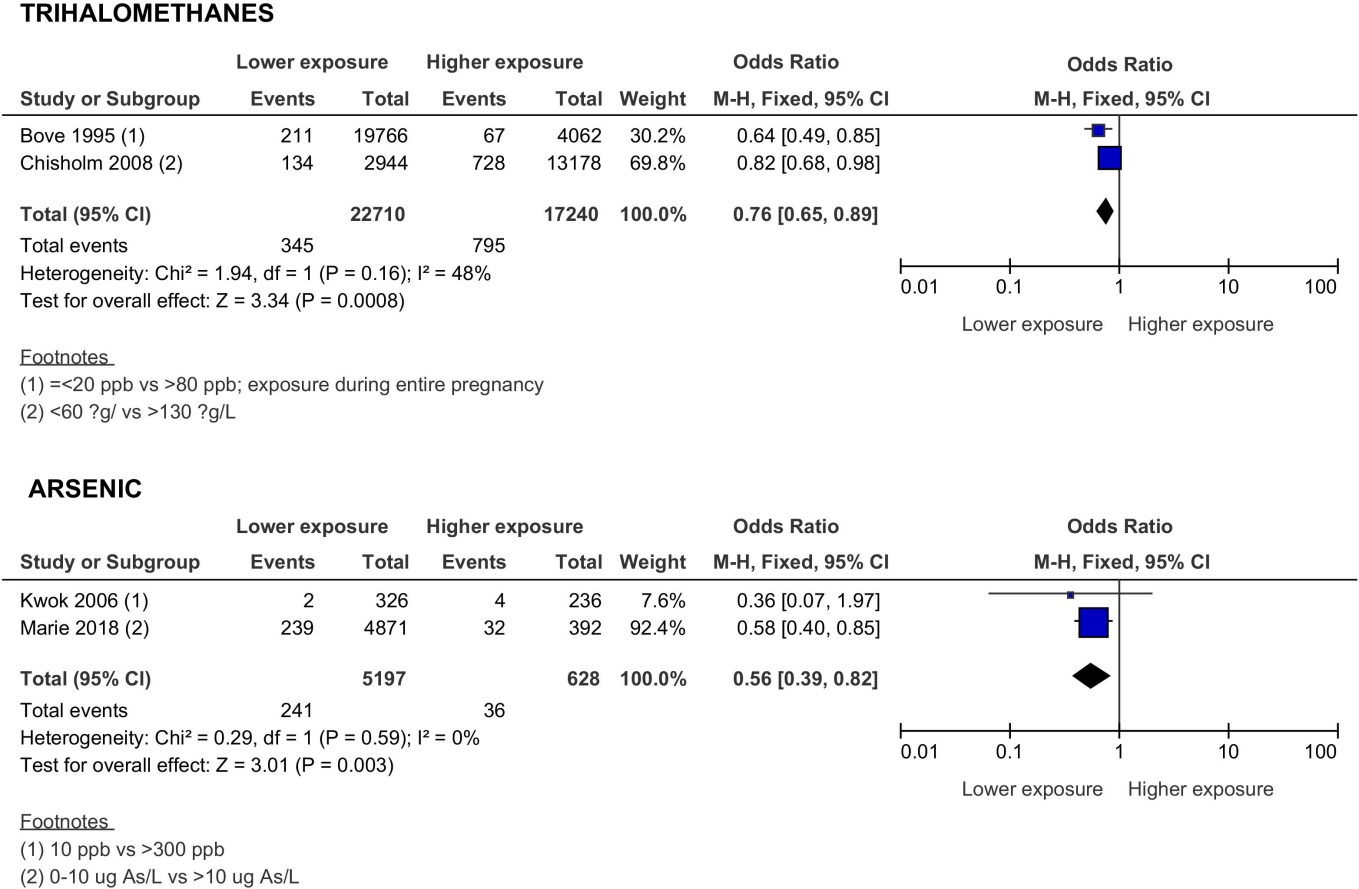
Pooled estimated and forest plots for total trihalomethanes and arsenic at lower and higher exposure during pregnancy and major birth defects.

Figure 2 presents the association between exposure to THMs and arsenic during pregnancy and the incidence of major birth defects, at low and high exposures. Both pollutants were associated with a lower risk of major birth defects at lower exposures compared with higher exposure (OR 0.76, 95% CI 0.65 to 0.89 and OR 0.56, 95% CI 0.39 to 0.82, respectively).

## Discussion

### Main findings

The detailed data analysis of the 32 studies included in this systematic review shows that 21 studies reported an association between a panel of 17 different pollutants in drinking water distributed by local water companies and an increased risk or incidence of overall non-syndromic birth defects and/or specific birth defect (table 1). We found evidence of an association between maternal exposure levels to TTMs and arsenic and an increase in major birth defects at high exposures.

### Comparison with previous studies

Birth defects occur in approximately 1 in 33 newborns in the USA and are estimated to affect around 8 million babies worldwide each year.^49^ Non-syndromic or isolated birth defects account for up to 75% of all birth defect cases and the most prevalent malformations, that is, CHDs, NTDs, OFDs and limb defects.^50^ The aetiopathology of individual birth defects remains unknown in 70% of the cases.^51^ A relatively small proportion of birth defects can be attributed, at least in part, to specific environmental causes such as congenital viral or parasitic infections and the use of pharmaceuticals (eg, valproic acid) or recreational drugs (eg, cocaine) in early pregnancy. However, the majority of birth defects are considered the result of multiple environmental factors acting together with an individual’s genetic susceptibility.^14^ This can also explain the wide variation in the incidence of overall or specific birth defects following exposure to the same water pollutant such as trichloroethane, THMs or arsenic (table 2). Similar heterogeneity in outcome data has been found for the association between congenital anomalies and maternal exposure to a variety of air pollutants during pregnancy.^52 53^

Globally, there are currently over 350 000 chemicals and mixtures of chemicals registered for production and use in the manufacturing industry, agriculture, food packaging, cosmetics and production industries among others.^54^ A large number of these chemicals have been registered only in LRCs and there are at least 900 pesticide, biocide and cosmetic active ingredients that are not covered by chemical inventories.^54^ The impact of many of these chemicals and unintentionally produced chemicals such as unreacted intermediates, by-products and degradation products on human health as well as on their releases, persistence, mobility in soil and rivers, and environmental fate are still unknown.

### Implications for clinical practice

The most used chemicals that have been investigated in observational studies, as shown in the present systematic review, are THMs, arsenic and nitrates (online supplemental table 1).

THMs are drinking water DBP that form when chlorine reacts with the organic matter in water^31^ and include mainly chloroform, bromodichloromethane, dibromochloromethane and bromoform. Chlorination of drinking water has been essential in eliminating waterborne infectious diseases in the Western world.^55^ THMs have been linked to small for gestational age (SGA) fetuses^56 57^ and may have carcinogenic effects^58^ but the evidence for both outcomes remains limited. A systematic review and meta-analysis of articles published up to December 2008 found an increased in overall birth defects (OR 1.17; 95% CI 1.02, 1.34) and in particular for ventricular septal defects (OR 1.58; 95% CI 1.21 to 2.07) associated for high versus low exposure to water chlorination during pregnancy, however, this meta-analysis was based on only three studies.^57^ The results of the present meta-analysis suggest an exposure level–response relationship (figure 2). A recent prospective large cohort study of 623 468 newborns has reported a decreased risk of CHDs after TTMs exposure during pregnancy,^47^ highlighting the heterogeneity of currently available data.

In Europe and North America, arsenic level in drinking water is regulated by national and international environment agencies and the WHO recommends concentration of arsenic of <10 µg/L.^58^ In many low-income and middle-income countries in Asia and South America, in part due to mining activities and the use of arsenic-based pesticides, arsenic levels in drinking water often exceed >300 µg/L and have been associated with mass poisoning.^59^ Chronic arsenic exposure has been associated with an increased risk of developing type 2 diabetes, cardiovascular diseases and cancer.^60^ Similar relationships exist with other heavy metals such as lead, mercury and cadmium. The most recent article identified in our systematic review was a retrospective case–control study of data collected in the USA between 2003 and 2008 (table 1) which, reported no association between birth defects and arsenic, cadmium or lead.^37^ However, like for TTMs exposure, the present data suggest an exposure level–response relationship for the overall risk of major birth defects (figure 2).

Nitrate and nitrite ions are widespread in the environment and are found naturally in plants and water.^2^ However, their increasing use in inorganic fertiliser and as additives in processed food has led to a global increase in nitrate levels in water resources. High levels have been associated with abnormal pregnancy outcomes, thyroid disease, risk of specific cancers, that is, colorectal, breast and bladder cancer.^61^ A recent systematic review and meta-analysis of articles published up to November 2022 on the association between nitrate in drinking water and adverse reproductive outcomes found an increased risk of preterm birth risk of NTDs based on the data of three cohort and two case–control studies, respectively.^62^ Two large recent Scandinavian cohort studies, published after the above systematic review, reported an increased risk of SGA for a median exposure <25 mg/L but not for an exposure >25 mg/L.^48 63^ No increased risk of overall birth defects was also reported, however, the authors observed a higher incidence of eye defects.^48^ Together, this highlights the inconsistency in the data available on nitrates exposure and pregnancy outcomes.

The teratogenic effects of any chemicals are the consequence of an insult between from day 31 after the last menstrual period in a 28-day cycle to 71 days from the last period and thus depend on the ability of the corresponding molecule to cross the placental barrier during that period.^51 60^ The use of laboratory animal models such as rodents to study placental transfer of water pollutants is limited due to species differences in placental biological functions, transporters, molecular kinetics and metabolism. The transfer of heavy metals by the human placenta has been extensively studied following the 1958 Minamata disaster.^12 64^ These studies found that methylmercury easily crosses the placental barrier compared with lead, arsenic and cadmium.^65^,^67^ Yet, methylmercury is not regarded as a teratogen in the conventional sense as it did not cause structural congenital birth defects.^12^ Furthermore, this contamination did not occur via drinking water but was the consequence of the maternal diet which included mainly fish and shellfish contaminated by methylmercury from the effluent of a plastic plant in Minamata Bay. There are very few studies that investigate the placental transfer of other drinking water pollutants such as THMs or nitrates.^68 69^ In only 1 of the 32 studies included in the present review did the authors present data on maternal serum levels of arsenic.^38^ Future prospective studies comparing maternal levels during all three trimesters of pregnancy and cord blood levels at birth are needed to better understand the placental transfer of water pollutants and accurately evaluate individual fetal exposure to drinking water pollutants. New statistical methodologies^70 71^ should be considered when examining the link between water pollutants and health outcomes in general and perinatal outcomes in particular.

### Strengths and limitations

This is the largest systematic review examining the possible association between the different drinking water pollutants reported in the international literature and non-syndromic birth defects. We performed a broad search for all known common drinking water pollutants and all the studies included in our systematic review provided secure medical records, regional or national databases with detailed descriptions of all birth defects in a defined population with detailed pathology records when required.

The main limitation of this study is the many challenges in assessing prenatal exposure to environmental pollutants in general and the many contaminants in water that can be found in different concentrations in water samples at any one time in particular.^72^ The studies included in our systematic review had varied designs including differences in timing and duration of exposures to a drinking water pollutant before and during pregnancy. The authors also used different methodologies in the evaluation of the concentration of the different pollutant components and different ranges for individual pollutant levels with different regulatory limits in different countries. Another limitation of this kind of study is a possible over-reliance on database studies that focus on correlations rather than causal links.^72^ 12 of the cohort studies^2021 23^,^25 29^ and 1 of the case–control study^37^ included in our systematic review used large population-level registers. These large databases are unable to provide data for confounding factors associated with birth defects such as individual work-related exposure to high levels of different water and air pollutants and other environmental toxins, folic acid supplementation before and during pregnancy,^73^ incidence of uncontrolled type 1 diabetes and the use of pharmaceutical medications and drug abuse. Only two studies provided data on individual maternal serum or urine pollutant measurements during pregnancy.^34 38^ In addition, all studies except one were conducted retrospectively, which limits the use of a standard meta-analysis to compare the data of most studies currently published in the international literature.^74^

### Conclusions

Evaluation of any links between birth defects and environmental exposures is likely to be limited due to constraints of quality and availability of data for exposure to a single water pollutant. Overall, the potential teratogenic effects of a specific chemical molecule have specific and narrow critical periods of susceptibility that may span only days, and considerably depend on exposure doses and placental transfer mechanisms. Animal models have been the gold standard to obtain teratogenic data, but interspecies differences have limited the suitability of those models. The evidence of an association between exposure to average levels of common drinking water chemical pollutants during pregnancy and an increased risk or incidence of birth defects is sparse and often contradictory. There is only evidence that any of the current common chemical water pollutants have a direct teratogenic effect on the developing human fetus at higher maternal exposure levels, such as, in case of professional exposure. These findings may help to advise patients about the risk of birth defects following exposure to common drinking water pollutants during pregnancy and to design further prospective studies using standardised research protocol.

## supplementary material

10.1136/bmjopen-2024-084122 online supplemental file 1

10.1136/bmjopen-2024-084122 online supplemental file 2

10.1136/bmjopen-2024-084122 online supplemental file 3

## Data Availability

Data are available on reasonable request.
