# Supplementary material for: Exposure to drinking water pollutants and non-syndromic birth defects: a systematic review and meta-analysis synthesis
Source: BMJ Open. 2024 Nov 11;14(11):e084122. doi: 10.1136/bmjopen-2024-084122 (PMC11555108; doi:10.1136/bmjopen-2024-084122)
Supplement: online supplemental file 1 [file bmjopen-14-11-s001.docx]

**Online supplemental Appendix 1** Electronic search strategy

***Time period*:** January 1962 to April 2023

***Inclusion Criteria***

- Prospective or retrospective cohort, population studies and case-control studies that provided data on exposure to drinking water pollutants around conception or during pregnancy and non-syndromic birth defects.
- Original publications with data on the number of patients exposed and controls with incidence of total births defects or category of specific birth defects from pathology reports.

***Exclusion Criteria***

- Reviews, opinions, letters, protocols and conference proceedings.
- Articles including syndromic birth defects.
- Articles published before 1962.
- Articles in languages other than English.
- Non-human studies.
