# Supplementary figures and images for: Exposure to drinking water pollutants and non-syndromic birth defects: a systematic review and meta-analysis synthesis
Source: BMJ Open. 2024 Nov 11;14(11):e084122. doi: 10.1136/bmjopen-2024-084122 (PMC11555108; doi:10.1136/bmjopen-2024-084122)

**On line supplemental Figure 1:** Quality assessment using the Newcastle-Ottawa Scale.

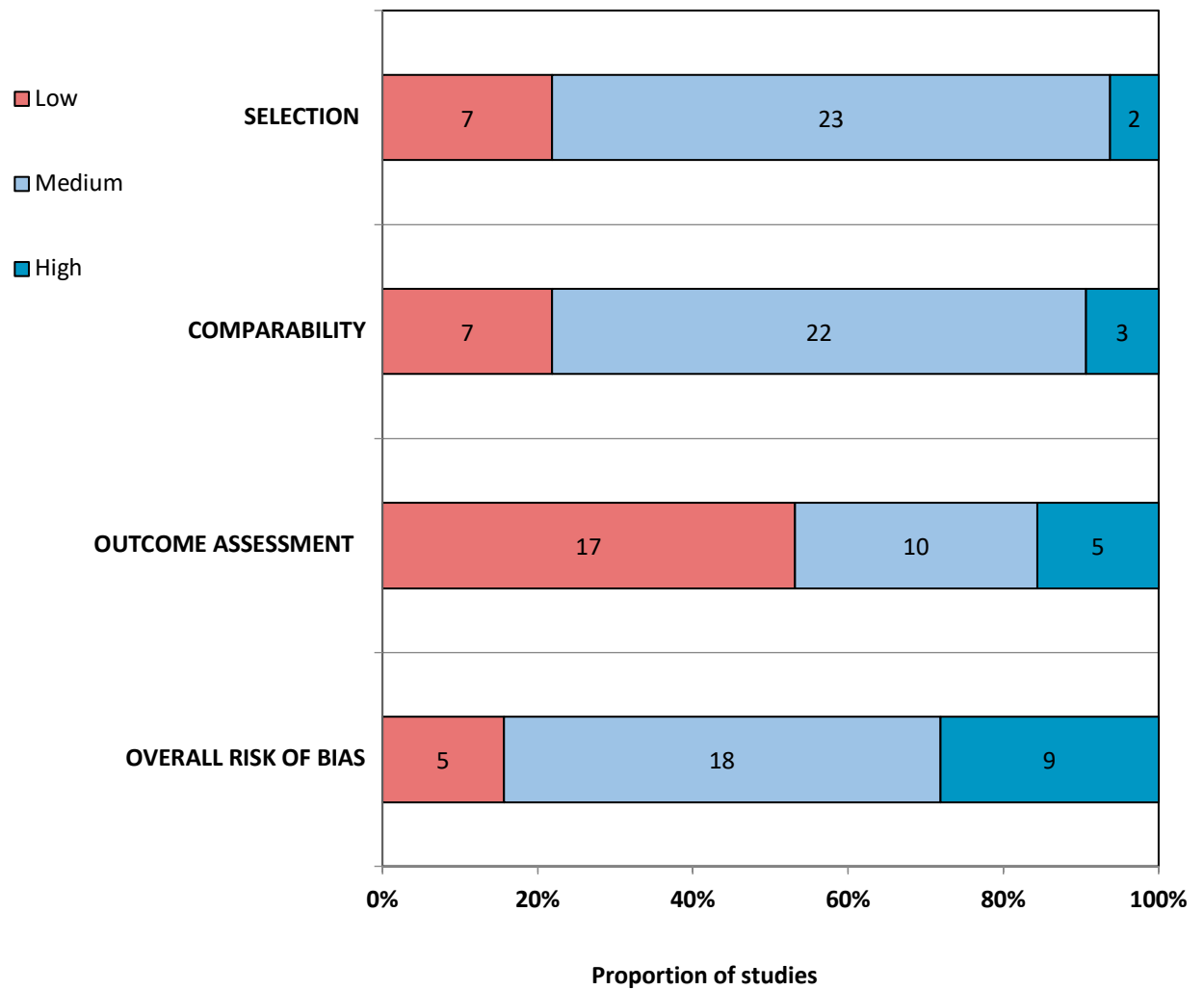

Supplement: online supplemental file 2 [file bmjopen-14-11-s002.pdf]
