# Supplementary material for: Exposure to drinking water pollutants and non-syndromic birth defects: a systematic review and meta-analysis synthesis
Source: BMJ Open. 2024 Nov 11;14(11):e084122. doi: 10.1136/bmjopen-2024-084122 (PMC11555108; doi:10.1136/bmjopen-2024-084122)
Supplement: online supplemental file 3 [file bmjopen-14-11-s003.docx]

**On line supplemental Table 1:** Association between birth defects and common drinking water pollutant exposure

**TRIHALOMETHANES**

| **Birth defect** | **Author et al. (Year)** | **Population studied (n)** | **Exposure** | **Association OR (%CI)** |
| --- | --- | --- | --- | --- |
| Overall BDs | Bove, et al. (1995)^23^  Chisholm, et al. (2008)^29^  Grazuleviciene, et al. (2013)^36^ | 81,523 births  20,870 births and TOPs  3074 live births | THMs at 80-100 ppb  THMs ≥ 130 μg/L  THMs 0.356–2.448 μg/d^*^ | OR 1.57 (50%CI 1.42;1.75)  OR 1.22 (95%CI 1.01;1.48)  OR 2.16 (95%CI 1.05;4.46) |
| All CHDs  Major CHDs  VSDs | Dodds, et al. (2001)^25^  Chisholm, et al. (2008)^29^  Nieuwenhuijsen et al (2008)^31^  Grazuleviciene, et al. (2013)^36^  Säve-Söderbergh, et al. (2021)^47^  Hwang et al (2008)^30^ | 49,842 live births  20,870 births and TOPs  2,605,226 births and TOPs  3074 live births  623,468 newborns  396,049 births | BDCM >20 μg/l  THMs ≥ 130 μg/L  THMs ≥ 60 μg/L  Bromoform ≥ 4 μg/L  THMs 0.356–2.448 μg/d^*^  THMs >15 μg/l  THMs >20 μg/l | RR 0.30 (95%CI 0.2;0.7)  OR 1.62 (95%CI 1.04;2.51)  OR 1.62 (95%CI 1.04;2.51)  OR 1.18 (95%CI 1.00;1.39)  OR 1.54 (95%CI 0.89;2.68)  OR 0.87 (95%CI: 0.77;0.99)  OR 1.81 (95%CI 0.98;3.35) |
| All NTDs  Anencephaly | Dodds, et al. (2001)^25^  Säve-Söderbergh, et al. (2021)^47^  Hwang et al (2008)^30^ | 49,842 live births  623,468 newborns  396,049 births | BDCM >20 μg/l  THMs >15 μg/l  THMs >20 μg/l | RR 2.5 (95%CI 0.67;2.10)  OR 1.82 (95%CI: 1.07;3.12  OR 1.96 (95%CI 0.94;4.07) |
| All OFDs  Cleft palate | Dodds, et al. (2001)^25^  Hwang et al (2008)^30^ | 49,842 live births  396,049 births | BDCM >20 μg/l  THMs >20 μg/l | RR 1.01 (95%CI 1.2;5.1)  OR 1.56 (95%CI 1.00;2.41) |
| UGDs | Grazuleviciene, et al. (2013)^36^  Säve-Söderbergh, et al. (2021)^47^ | 3074 live births  623,468 newborns | THMs 0.356–2.448 μg/d^*^  THMs >15 μg/l | OR 3.01 (95%CI 1.11;8.16)  OR 2:06 (95%CI 1.53;2.78) |
| Others defects  Gastroschisis  Musculoskeletal Limbs | Nieuwenhuijsen et al (2008)^31^  Grazuleviciene, et al. (2013)^36^  Säve-Söderbergh, et al. (2021)^47^ | 2,605,226 births and TOPs  3074 live births  623,468 newborns | Bromoform ≥ 4 μg/L  THMs 0.356–2.448 μg/d^*^  THMs >15 μg/l | OR 1.38 (95%CI 1.00;1.92)  OR 0.74 (95%CI 0.39;1.42)  OR 1:34 (95%CI:1.10; 1.64) |

**ARSENIC**

| **Birth defect** | **Author et al. (Year)** | **Population studied (n)** | **Exposure** | **Association OR (95%CI)** |
| --- | --- | --- | --- | --- |
| Overall BDs | Kwok, et al. (2006)^28^  Marie, et al. (2018)^41^ | 2,189 pregnancies  5263 pregnancies | >300 ppb  >10 μg/L | OR 1.005 (95% CI 1.001;1.010)  OR 2.41 (95%CI 1.36;4.14)^§^ |
| All CHDs | Marie, et al. (2018)^41^  Richter, et al. (2021)^46^ | 5263 pregnancies  1,042,413 live births | >10 μg/L  ≥5.0 μg/L | OR 3.66 (95%CI 1.62;7.64)^§^  OR 1.42 (95% CI 1.24;1.62) |

**NITRATES**

| **Birth defect** | **Author et al. (Year)** | **Population studied (n)** | **Exposure** | **Association OR (95%CI)** |
| --- | --- | --- | --- | --- |
| Overall BDs | Stayner et al (2022)^48^ | 1,018,914 live births | >10 mg/L | OR 0.93 (95%CI 0.88;0.99) |
| All NTDs | Blaisdell et al (2019)^44^ | 348,250 live births | > 25 mg/L | RR 1.03 (95% CI 0.84;1.27 |
| All OFDs | Blaisdell et al (2019)^44^ | 348,250 live births | > 25 mg/L | RR 0.93 (95%CI 0.82;1.06) |
| Others defects  Limbs Gastroschisis  Hypospadias  Ocular | Blaisdell et al (2019)^44^  Stayner et al (2022)^48^ | 348,250 live births  1,018,914 live births | > 25 mg/L  > 25 mg/L  > 25 mg/L  >10 mg/L | RR 1.26 (95%CI 1.05;1.51)  RR 0.98 (95%CI 0.91;1.06)  RR 0.94 (95%CI 0.76;1.16)  OR 1.29 (95%CI 1.00;1.66) |

^*^tertiles for the average TTHM concentration; ^§^female newborns only when adjusted for gender

HAAs= Halocetic acids; THMs= Trihalomethanes; TTHM= Total Trihalomethane.

BDs= Birth defects; CHDs= Congenital heart defects; NTDs= Neural tube defects; OFDs: Orofacial defects; UGDs= urogenital defects; VSDs= ventricular septal defects.

OR= Odd ratio; RR= Relative risk.
