# Supplementary material for: Exposure to drinking water pollutants and non-syndromic birth defects: a systematic review and meta-analysis synthesis
Source: BMJ Open. 2024 Nov 11;14(11):e084122. doi: 10.1136/bmjopen-2024-084122 (PMC11555108; doi:10.1136/bmjopen-2024-084122)
Supplement: Supplementary file 4 [file bmjopen-2024-084122.reviewer_comments.pdf]

PEER REVIEW HISTORY

BMJ Open publishes all reviews undertaken for accepted manuscripts. Reviewers are asked to complete a checklist review form and are provided with free text boxes to elaborate on their assessment. These free text comments are reproduced below.

ARTICLE DETAILS

Title (Provisional)

Exposure to drinking water pollutants and non-syndromic birth defects: a systematic review and meta-analysis synthesis

Authors

Jauniaux, Eric; Jeremiah, Lydia; Richardson, Biba; Rogozińska, Ewelina

---

VERSION 1 - REVIEW

**Reviewer**	**1**
**Name**	**Damania, Richard**
**Affiliation**	**World Bank**
**Date**	**26-Jan-2024**
**COI**	**None.**

Thank you for inviting me to review this excellent paper that is written with clarity and executed with competence. The paper clearly addresses an important health issue with potentially wide and uncertain economic ramifications. As such my comments are minor and relate to the discussion and next steps rather than the conduct of the meta-analysis. It is hoped that some of the comments are helpful in revising the paper.

The results of the review are somewhat disappointing as there are no clear and robust effects that emerge from the review. The paper rightly acknowledges the many shortcomings of the existing literature. Perhaps it would be helpful to take this a step further and discuss how future analyses could address these deficiencies by employing more recent statistical techniques that seek to establish causality where possible.

For instance, the paper notes that the majority of studies controlled for standard (demographic) confounders. It is, however, widely acknowledged that it is impossible to control for all possible confounders, hence exclusion restrictions will be violated compromising the accuracy and interpretation of the results. As an obvious example, households who consume “safer” bottled water or in possession of RO filters will likely be immune to impacts. If there is no data on this for controls, estimates and SEs will be biased for known reasons. Such problems are common in

statistics and econometrics, hence researchers use time and (say) household level fixed effects to account for all of the unobservable that may change over time and across individuals. It is unclear if any of the studies do this and if so it would suggest greater accuracy (all else equal).

A related problem is that there are usually multiple pollutants and most are unmeasured as the paper acknowledges. Hence we cannot be certain if effects are from the focal pollutants or their synergistic (compounding) impacts with an unmeasured pollutant.

Given these and other related challenges in identifying causal impacts a variety of new methods have been developed such as regression discontinuity design (RDD) or where interventions are "unique" synthetic control methods and other approaches. The paper would benefit from a discussion of the limits of the current literature and how these familiar problems can be addressed.

Reviewer 2

Name Zahid, Muhammad

Affiliation University of Nebraska Medical Center, Environmental, Agricultural, and Occupational Health

Date 02-Feb-2024

COI I have no competing interest.

Reviewer comments

Manuscript Number: **BMJOPEN-2024-084122**

Manuscript Title: Exposure to drinking water pollutants and non-syndromic birth defects: A systematic review and meta-analysis synthesis

Recommendation: **Accepted after minor revision**

This systematic review and meta-analysis manuscript analyzes the possible association between drinking water contaminants and non-syndromic birth defects. The authors articulate the research question and analyze the data from 32 studies in a very sound manner. Interestingly, the authors only found some association of overall birth defects with TTMs exposure level-response. However, it would be good if the authors discussed other chemicals that are considered hazardous regarding birth defects, for example, atrazine. In addition, some suggestions should be addressed before the final acceptance. For example

Page 3: Add a colon after each bold title for example
Design: Systematic review and meta-analysis synthesis

Article Summary:

This is the largest systematic —This is the most extensive systematic

between know common drinking water pollutants, different drinking water pollutants between

detectable drinking water pollutants

pathology record --- pathology records

environment pollution environmental pollution

of the each pollutant components-- of each pollutant component

Introduction

defects from early in life defects from early life or defects from childhood

and the developmental anomalies and developmental anomalies

Data source and search strategy:

including following MeSH including the following MeSH

lists of from full-text articles lists of full-text articles.

The above sentence has relevant (2 times) in the same sentence better rewrite

Selection criteria---:

Two reviewers (L.J and B.R) independently assessed identified titles and abstracts against the eligibility criteria.

The study had to meet following criteria in order to be included –The study had to meet the following criteria to be included

case control---case-control (CHECK ALL PLACES)

editorial—editorials

case reports---case reports,

same centres or with indication-----same centers or with an indication

As our aim was to----As we aimed to evaluate

The last paragraph seems repeated; I suggest better moving these lines and combining them with the first line.

Data Synthesis

metaanalysis -- meta-analysis

were not included in meta-analysis --- were not included in the meta-analysis

Patient and public involvement

question, and in developing plans for design, -- question and in developing plans for the design

Data search results

supplemental Figure-- Supplemental Figure

Characteristics of studies included in the systematic review

local water provider matched with individual home address-- local water providers matched with individual home addresses

used timing—used the timing

national environment agency----National Environment Agency

Main finding

reported on an association----reported an association

17 different pollutants present in drinking water--17 different pollutants in drinking water (any figure reference where 17 pollutants named mentioned)

evidence of association----evidence of an association

i.e ----i.e.,

e.g ---e.g.,

Implications for clinical practice

The most used chemicals and which have been—The most used chemical and that have

been and include mainly --- and includes mainly

water borne ---- watreborne

infectious disease in the Western ---- infectious diseases in the Western

outcomes remain limited----- outcomes remains limited

North-America----North America

South-America --- South America

and the used arsenic-based -- and arsenic-based

three cohort--- three cohorts

Overall birth defect was----overall birth defects were

methylmercury crosses easily----methylmercury easily crosses

consequence of maternal diet which---- consequence of the maternal diet, which

When considering the international--different regulatory limits in different countries ----- too long sentence
and many times different used better break it in two and rewrite in a better

way Table 1, Page 28, Population column, row 24: cases with a BD

**Reviewer**	**3**
**Name**	**Xu, Man**
**Affiliation**	**Hubei University of Chinese Medicine**
**Date**	**08-Jul-2024**
**COI**	**No.**

I have conducted a comprehensive review of your paper and provided constructive feedback aimed at enhancing the quality and impact of your article. I encourage you to carefully consider these suggestions to improve clarity and overall effectiveness. Once revisions are implemented, I look forward to reviewing the updated version. Your dedication to refining your work is commendable, and I am eager to witness the evolution of your article. Best regards.

Abstract

1. The discussion section seems to include the primary results of your study, but the abstract presented does not fully reflect these main findings. Specifically, the abstract should detail the levels of exposure to different pollutants and the types of birth defects observed, as well as any notable statistical associations found.

2. Clearly report the significance level of the statistical analysis, such as a p-value, to support the confidence of the conclusions.

3. The conclusion should elaborate on the practical implications and applications of the findings, particularly their impact on public health policy.

Introduction

1. The introduction provides a good overview, but it should be more detailed explanations of the specific pollutants and their known effects on human health.

2. To strengthen the argument for the study's necessity, focus on summarizing recent and directly relevant studies that investigate the relationship between drinking water pollutants and birth defects. Highlight the inconsistencies or gaps in these studies to justify the need for your research.

3. Clearly articulate the gaps in current knowledge and how your study aims to address them. Emphasize the public health implications of filling these gaps, particularly for vulnerable populations such as pregnant women.

4. Incorporate specific examples and statistical data to make the argument more compelling. Include data on the prevalence of birth defects in relation to water pollution.

Methods

1. The criteria for including studies in the meta-analysis and the methods used for data synthesis need further elaboration. Explain how studies were assessed for comparability and how heterogeneity was addressed in the meta-analysis.

Results

1. The quality assessment indicates that only five studies had a low risk of bias, with the majority having a moderate risk. Discuss how this was accounted for in the synthesis of the results.

2. Clearly report the significance level of the statistical analysis, such as a p-value, to support the confidence of the conclusions.

3. Consider providing the results of a sensitivity analysis to demonstrate the robustness of the results.

Discussion

1. While the main findings are summarized, the section could be more concise and focused. A clear and succinct summary of the key findings with less repetition would improve readability.

2. Elucidate the biological mechanisms underlying the observed associations. Specifically, explain how THMs, arsenic, and nitrates impact fetal development through their respective biological pathways to enhance the depth and persuasiveness of the discussion.

3. Emphasize the practical applications of the findings and potential policy changes. Expand on how these findings can inform clinical guidelines and public health policies.

4. Clearly outline specific areas for future research, including prospective studies, investigations into other pollutants, and mechanisms of action.

VERSION 1 - AUTHOR RESPONSE

Reviewer: 1
Richard Damania, World Bank
Comments to the Author:
Thank you for inviting me to review this excellent paper that is written with clarity and executed with competence. The paper clearly addresses an important health issue with potentially wide and uncertain economic ramifications. As such my comments are minor and relate to the discussion and next steps rather than the conduct of the meta-analysis. It is hoped that some of the comments are helpful in revising the paper.
We thank the reviewer for his constructive comments.

The results of the review are somewhat disappointing as there are no clear and robust effects that emerge from the review. The paper rightly acknowledges the many shortcomings of the existing literature. Perhaps it would be helpful to take this a step further and discuss how future analyses could address these deficiencies by employing more recent statistical techniques that seek to establish causality where possible.

For instance, the paper notes that the majority of studies controlled for standard (demographic) confounders. It is, however, widely acknowledged that it is impossible to control for all possible confounders, hence exclusion restrictions will be violated compromising the accuracy and interpretation of the results. As an obvious example, households who consume “safer” bottled water or in possession of RO filters will likely be immune to impacts. If there is no data on this for controls, estimates and SEs will be biased for known reasons. Such problems are common in statistics and econometrics, hence researchers use time and (say) household level fixed effects to account for all of the unobservable that may change over time and across individuals. It is unclear if any of the

studies do this and if so it would suggest greater accuracy (all else equal).

A related problem is that there are usually multiple pollutants and most are unmeasured as the paper acknowledges. Hence we cannot be certain if effects are from the focal pollutants or their synergistic (compounding) impacts with an unmeasured pollutant.

Given these and other related challenges in identifying causal impacts a variety of new methods have been developed such as regression discontinuity design (RDD) or where interventions are "unique" synthetic control methods and other approaches. The paper would benefit from a discussion of the limits of the current literature and how these familiar problems can be addressed.

The issue raised by the reviewer are relevant but beyond the scope of this paper. The objective of our review was to summarise the literature on the link between drinking water pollutants and non-syndromic birth defects. We recognised the impact of study design and data of the articles included in our systematic review on our findings and have suggested that these methods should be evaluated by new prospective studies and added the following new references (70 & 71)

Piel FB, Fecht D, Hodgson S, Blangiardo M, Toledano M, Hansell AL, Elliott P. Small-area methods for investigation of environment and health. Int J Epidemiol. 2020;49:686-699.

Popovic M, Zugna D, Tilling K, Richiardi L. Regression discontinuity design for the study of health effects of exposures acting early in life. Front Public Health. 2024;12:1377456.

Reviewer: 2
Dr. Muhammad Zahid, University of Nebraska Medical Center
Comments to the Author:
This systematic review and meta-analysis manuscript analyzes the possible association between drinking water contaminants and non-syndromic birth defects. The authors articulate the research question and analyze the data from 32 studies in a very sound manner. Interestingly, the authors only found some association of overall birth defects with TTMs exposure level-response. However, it would be good if the authors discussed other chemicals that are considered hazardous regarding birth defects, for example, atrazine.

In addition, some suggestions should be addressed before the final acceptance. For example:

Page 3: Add a colon after each bold title for example
Design: Systematic review and meta-analysis synthesis
A space was added after each bod subtitles as per the design of BMJ open articles

Article Summary:
This is the largest systematic —This is the most extensive systematic
between know common drinking water pollutants, different drinking water pollutants ----
between detectable drinking water pollutants
pathology record--- pathology records
environment pollution --- environmental pollution
of the each pollutant components-- of each pollutant component

Introduction
defects from early in life--- defects from early life or defects from childhood
and the developmental anomalies--- and developmental anomalies
Data source and search strategy:
including following MeSH--- including the following MeSH
lists of from full-text articles--- lists of full-text articles.
The above sentence has relevant (2 times) in the same sentence better rewrite
Selection criteria---:
Two reviewers (L.J and B.R) independently assessed identified titles and abstracts against the eligibility criteria.
The study had to meet following criteria in order to be included –The study had to meet the following criteria to be included
case control---case-control (CHECK ALL PLACES)
editorial—editorials
case reports---case reports,
same centres or with indication---- same centers or with an indication
As our aim was to----As we aimed to evaluate
The last paragraph seems repeated; I suggest better moving these lines and combining them with the first line.

Data Synthesis
metaanalysis---meta-analysis
were not included in meta-analysis--- were not included in the meta-analysis
Patient and public involvement
question, and in developing plans for design, -- question and in developing plans for the design

Data search results
supplemental Figure-- Supplemental Figure

Characteristics of studies included in the systematic review
local water provider matched with individual home address-- local water providers matched with individual home addresses
used timing—used the timing
national environment agency--- National Environment Agency

Main finding
reported on an association--- reported an association
17 different pollutants present in drinking water--17 different pollutants in drinking water (any figure reference where 17 pollutants named mentioned)
evidence of association--- evidence of an association
i.e.--- i.e.,
e.g.---e.g.,

Implications for clinical practice
The most used chemicals and which have been—The most used chemical and that have been

and include mainly --- and includes mainly
water borne ----watreborne
infectious disease in the Western --- infectious diseases in the Western
outcomes remain limited---- outcomes remains limited
North-America--- North America
South-America--- South America
and the used arsenic-based---and arsenic-based
three cohort--- three cohorts
Overall birth defect was ---overall birth defects were
methylmercury crosses easily--- methylmercury easily crosses
consequence of maternal diet which--- consequence of the maternal diet, which
Table 1, Page 28, Population column, row 24: cases with a BD
We thank the reviewer for spotting the above typos which were corrected accordingly.

When considering the international--different regulatory limits in different countries.----too long sentence and many times different used better break it in two and rewrite in a better way.
Modified as suggested.

<u>Reviewer: 3</u>
Dr. Man Xu, Hubei University of Chinese Medicine
Comments to the Author:
I have conducted a comprehensive review of your paper and provided constructive feedback aimed at enhancing the quality and impact of your article. I encourage you to carefully consider these suggestions to improve clarity and overall effectiveness. Once revisions are implemented, I look forward to reviewing the updated version. Your dedication to refining your work is commendable, and I am eager to witness the evolution of your article. Best regards.
We thank the reviewer for his constructive comments.

Abstract
1. The discussion section seems to include the primary results of your study, but the abstract presented does not fully reflect these main findings. Specifically, the abstract should detail the levels of exposure to different pollutants and the types of birth defects observed, as well as any notable statistical associations found.
2. Clearly report the significance level of the statistical analysis, such as a p-value, to support the confidence of the conclusions.
3. The conclusion should elaborate on the practical implications and applications of the findings, particularly their impact on public health policy.
The abstract was modified according to the above comments including additional results, P values and suggestions for future studies.

Introduction
1. The introduction provides a good overview, but it should be more detailed explanations of the specific pollutants and their known effects on human health.
2. To strengthen the argument for the study's necessity, focus on summarizing recent and directly relevant studies that investigate the relationship between drinking water pollutants

and birth defects. Highlight the inconsistencies or gaps in these studies to justify the need for your research.
3. Clearly articulate the gaps in current knowledge and how your study aims to address them. Emphasize the public health implications of filling these gaps, particularly for vulnerable populations such as pregnant women.
4. Incorporate specific examples and statistical data to make the argument more compelling. Include data on the prevalence of birth defects in relation to water pollution.
These points are all included in the discussion.

Methods
1. The criteria for including studies in the meta-analysis and the methods used for data synthesis need further elaboration. Explain how studies were assessed for comparability and how heterogeneity was addressed in the meta-analysis.
We did not really address the heterogeneity. Our approach was pragmatic. Meta-analysis of observational studies is frequently limited due to the nature of the study designs which was the case in the present systematic review.

Results
1. The quality assessment indicates that only five studies had a low risk of bias, with the majority having a moderate risk. Discuss how this was accounted for in the synthesis of the results.
2. Clearly report the significance level of the statistical analysis, such as a p-value, to support the confidence of the conclusions.
3. Consider providing the results of a sensitivity analysis to demonstrate the robustness of the results.
Figure 1 (Pooled estimated and forest plots for total trihalomethanes and arsenic at lower and higher exposure during pregnancy and major birth defects) presents 95% confidence intervals and p-values for the corresponding analysis. We had insufficient number of studies to perform sensitivity analyses.

Discussion
1. While the main findings are summarized, the section could be more concise and focused. A clear and succinct summary of the key findings with less repetition would improve readability.
The main findings are summarized in on short paragraph with no repetition.
2. Elucidate the biological mechanisms underlying the observed associations. Specifically, explain how THMs, arsenic, and nitrates impact fetal development through their respective biological pathways to enhance the depth and persuasiveness of the discussion.
This is well beyond the scope of this type of study and having reviewed the literature in details, we found no clear mechanistic descriptions for the 17 different pollutants reported in or study. This is also the case for the vast majority of medical drugs and the main limitation is the use of laboratory animal models that do not reflect difference in placental biology and embryogenesis. A sentence referring to this issue was added to the discussion and conclusion.
3. Emphasize the practical applications of the findings and potential policy changes. Expand on how these findings can inform clinical guidelines and public health policies.

4. Clearly outline specific areas for future research, including prospective studies, investigations into other pollutants, and mechanisms of action.
As per the comments of reviewer 1, we have added a sentence and 2 references to support further studies. We have also made changes through the discussion to add to the above points.

animal models have been the gold standard to obtain teratogenic data, but inter-species differences have limited the suitability of those models

---

VERSION 2 - REVIEW

**Reviewer**	**1**
**Name**	**Damania, Richard**
**Affiliation**	**World Bank**
**Date**	**23-Sep-2024**
**COI**	

This is a useful paper that is written with clarity. As implied in an earlier review the authors should not simply assert in a single sentence that the statistical methods are inadequate due to lack of control of confounders. Rather this has 2 clear implications. First that the conclusions of the studies are biased due to violations of the "exclusion condition". And we cannot a priori determine the direction of bias. Second the studies are correlations and statistically causal. This too weakens the interpretation.

In sum the evidence reviewed in this paper is a lot weaker than is stated here and would not make it to publication in other more statistically oriented disciplines.

**Reviewer**	**3**
**Name**	**Xu, Man**
**Affiliation**	**Hubei University of Chinese Medicine**
**Date**	**02-Oct-2024**
**COI**	

The manuscript presents an interesting investigation into the potential links between drinking water pollutants and non-syndromic birth defects. The study's objective is relevant and timely, given the growing concerns over environmental health. The research appears to

be methodologically sound, and the analysis seems to be appropriately conducted. However, the implications of these findings for public health policy should be more thoroughly explored.

VERSION 2 - AUTHOR RESPONSE

Reviewer: 1
Richard Damania, World Bank
Comments to the Author: This is a useful paper that is written with clarity. As implied in an earlier review the authors should not simply assert in a single sentence that the statistical methods are inadequate due to lack of control of confounders. Rather this has 2 clear implications. First that the conclusions of the studies are biased due to violations of the "exclusion condition". And we cannot a priori determine the direction of bias. Second the studies are correlations and statistically causal. This too weakens the interpretation. In sum the evidence reviewed in this paper is a lot weaker than is stated here and would not make it to publication in other more statistically oriented disciplines.

The objective of our work is to summarise the literature on the subject of interest and is as strong as the identified primary research. As our work does not focus on the statistical issues around the environmental exposures, we don’t feel the need of going into the details of limitations of the statistical analysis; however, we have added sentences recognising the limitations of applied methods. in the limitations section of the discussion, and indicate the limitations in the conclusion and a reference by the reviewer highlighting these important points and which was not published when we submitted our manuscript.
Damania R, Zaveri E. Hidden toxins: The effects of water quality on pregnancy and infant health. BJOG. 2024;131:535-537.

Reviewer: 3
Dr. Man Xu, Hubei University of Chinese Medicine
Comments to the Author:
The manuscript presents an interesting investigation into the potential links between drinking water pollutants and non-syndromic birth defects. The study's objective is relevant and timely, given the growing concerns over environmental health. The research appears to be methodologically sound, and the analysis seems to be appropriately conducted. However, the implications of these findings for public health policy should be more thoroughly explored.

We agree and we hope that our study will stimulate more epidemiologic research on this important topic with better standardised methodology protocols as highlighted by reviewer 1.

VERSION 3 - REVIEW

Reviewer 1

**Name**	**Damania, Richard**
**Affiliation**	**World Bank**
**Date**	**15-Oct-2024**
**COI**	

The revised paper adequately acknowledges the limitations of the correlational analyses that comprise the bulk of the studies.
